# An Experimental Study to Assess the Ecotoxicity of Warfarin and Tinzaparin on Meiobenthic Amphipods: Original Taxonomic Data from Saudi Arabia and Computational Modeling

**DOI:** 10.3390/toxics13040264

**Published:** 2025-03-31

**Authors:** Amal Lassoued, Fehmi Boufahja, Gabriel Plavan, Naoufel Ben Hamadi, Mohamed A. M. Ali, Walid Elfalleh, Riadh Badraoui, Hamdi Bendif, Amor Hedfi

**Affiliations:** 1 Coastal Ecology and Ecotoxicology Unit, Laboratory of Environment Biomonitoring, Faculty of Sciences of Bizerte, University of Carthage, Zarzouna 7021, Tunisia; amalassoued@gmail.com; 2 Biology Department, College of Science, Imam Mohammad Ibn Saud Islamic University (IMSIU), Riyadh 11623, Saudi Arabia; mamzaid@imamu.edu.sa (M.A.M.A.); wbelfallah@imamu.edu.sa (W.E.); hlbendif@imam.edu.sa (H.B.); 3 Department of Biology, Faculty of Biology, “Alexandru Ioan Cuza” University, Bvd. Carol I, No. 20A, 700505 Iasi, Romania; gabriel.plavan@uaic.ro; 4 Chemistry Department, College of Science, Imam Mohammad Ibn Saud Islamic University (IMSIU), P.O. Box 5701, Riyadh 11432, Saudi Arabia; nabenhamadi@imamu.edu.sa; 5 Department of Biology, University of Ha’il, Ha’il 45851, Saudi Arabia; ri.badraoui@uoh.edu.sa; 6 Department of Biology, College of Sciences, Taif University, P.O. Box 11099, Taif 21944, Saudi Arabia; o.zaied@tu.edu.sa

**Keywords:** anticoagulants, meiofauna, amphipods, ecotoxicity, computational modeling

## Abstract

In the current research, we examined the effects of warfarin (W1 = 5 mg/L and W2 = 25 mg/L) and tinzaparin (T1 = 5 mg/L and T2 = 25 mg/L) on meiofauna using microcosms. These microcosms were intentionally contaminated with both anticoagulants for one month. The findings indicated that nematodes and amphipods demonstrated the greatest resistance to the two anticoagulants evaluated. Specifically, the number of amphipods increased after exposure to the treatment that included T2. Following the separate introduction of each drug, amphipods displayed a taxonomic restructuring, with a more significant impact observed from T2 and T2W1. Results were derived from multivariate analyses of a compilation of sensitive amphipod taxa in response to tinzaparin and warfarin. In contrast, different species were identified as positive indicators for tinzaparin. Ultimately, the similarity between the control amphipod replicates and those subjected to mixed anticoagulants (T1W1 and T2W2) suggests that warfarin may have reduced the toxicity of tinzaparin. Additionally, the computational study indicated that warfarin interacts with 4XNN and forms strong molecular interactions with several key residues, which contribute to the toxicokinetic characteristics observed in the empirical findings.

## 1. Introduction

It is estimated that the global elderly population will reach two billion persons, accounting for approximately 22% of the total population. This increase in the elderly demographic has been associated with several blood disorders, including thrombosis, angiogenesis, inflammation, and tumor metastasis. Factors contributing to these conditions include aging, stress, a lack of physical activity, and poor nutritional habits. Consequently, there is a growing reliance on anticoagulant medications. According to recent studies, heparinoids such as tinzaparin and warfarin are now frequently recommended to manage various arterial and venous thromboembolic conditions.

The presence of heparinoids in water bodies, resulting from runoff and wastewater discharge, presents a significant environmental concern. These substances must be transformed into more soluble forms in the body before they are excreted through urine. During the COVID-19 pandemic, patients on warfarin and tinzaparin were noted to have an increased susceptibility to arterial and venous thrombosis. Immune thrombosis plays a critical role in the progression of COVID-19, while infections caused by the SARS-CoV virus trigger inflammatory responses. The use of tinzaparin for preventive anticoagulation, administered at various dosages from low to high therapeutic levels, has demonstrated effectiveness in both the treatment and prevention of COVID-19 in affected patients.

Anticoagulants pose significant threats to human health and aquatic environments, particularly due to their presence in wastewater. The risks associated with these substances have increased because of inadequate treatment facilities, the rising use of anticoagulant medications, their easy accessibility, and the growing elderly population.

Previous research, as highlighted in [1], has pointed out the limited ecotoxicological investigations of heparinoids. Most studies have primarily focused on standard acute toxicity assessments and macrofauna as indicator organisms [2]. The potential impacts of these foreign substances on small, stationary benthic populations at the bottom of the marine food chain remain unclear. Unlike filter-feeding macrofauna, smaller organisms in the food web, such as bacteria, protists, and meiofauna, have not been studied extensively in ecotoxicology. Therefore, this experimental study used meiobenthic organisms as indicators to evaluate the ecotoxicological effects of anticoagulants. These organisms are abundant, possess diverse taxonomic and functional characteristics, have rapid reproductive cycles, and exhibit a wide range of tolerance or vulnerability to various foreign substances [3,4].

Infrequent research has been conducted on meiofauna in Saudi Arabia, with species identification remaining unaddressed. Additionally, no studies have explored how meiobenthic taxa respond to heparinoids. This study aimed to assess the harmful effects of tinzaparin and warfarin—both individually and in combination—on amphipods along the coast of Jeddah, Saudi Arabia, to fill this research gap. Notably, most internationally published research has focused on free-living nematodes, while meiobenthic crustaceans, particularly amphipods, have received little attention. This lack of focus has contributed to a decline in the number of qualified taxonomists specializing in this group. Furthermore, this experiment investigated whether the interactions between tinzaparin and warfarin were additive, synergistic, or antagonistic. Finally, computational methods were employed to analyze binding affinity, intermolecular interactions, and toxicokinetic properties.

## 2. Materials and Methods

### 2.1. Location of the Collection Field and Experimental Processing

**Figure 1:**
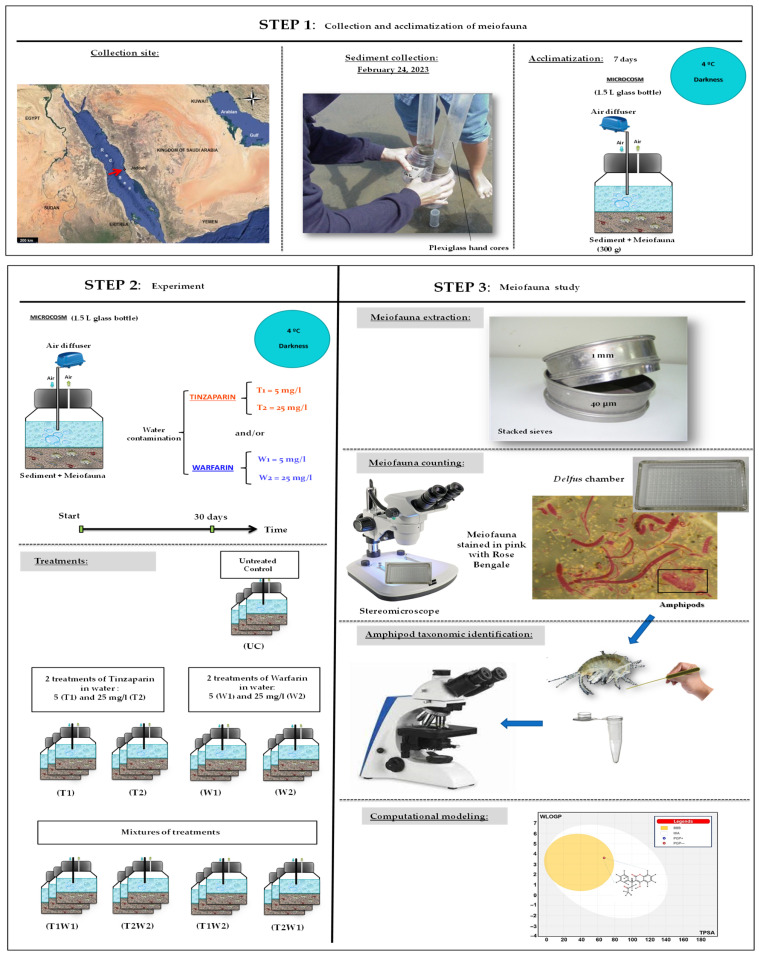
Graphical summary of steps and methodology adopted to experimentally study effects of tinzaparin and warfarin on meiofauna from Jaddah coasts, Saudi Arabia. The red arrow indicates the exact location of the collection site.

On 24 February 2023, at 7 a.m., sediment samples were collected from Al Saif Bay (21°14′0.07″ N, 39°8′43.13″ E), located near Jeddah, Saudi Arabia (see Figure 1). The top 5 cm of the sediment layer, taken from 50 cm beneath the surface, were gathered using Perspex hand cores with a 3.6 cm inner diameter and a 10 cm^2^ cross-section. A recent study identified the region as an unspoiled habitat, with the sediment primarily composed of sand (65.2%) and containing a minimal level of organic material (5.65%). This classification influenced the selection of sampling sites. A significant amount of meiofauna (485.2 ± 134.8 individuals, as reported in the study) was found in the sediment of this bay.

On the day of sampling, three abiotic factors were assessed at the sediment–water interface: the pH was measured using a WTW pH-meter model pH 330/SET-1, dissolved oxygen levels were recorded with a WTW oxymeter, and temperature and salinity were determined using a WTW LF 196 Thermo-Salinometer (produced in Weilheim, Germany).

The water content of the collected substrate was determined by drying it at 45 °C until a stable mass was reached. To analyze sediment particle sizes, the material was sieved with a water jet through 63 μm sieves and then dried at 45 °C. The average grain size was calculated using cumulative curves, and the total organic matter percentage was evaluated using the mass loss technique at 450 °Cover 6 h.

### 2.2. Choice of Warfarin and Tinzaparin Concentrations

To the best of our knowledge, there is no information available in the literature regarding the levels of warfarin and tinzaparin in seawater. This investigation commenced during the latter stages of the COVID-19 crisis. Consequently, it was anticipated that the concentrations of these two anticoagulants would be significant in coastal waters, which directly receive urban wastewater, particularly for low-molecular-weight heparins [5,6]. The recommended daily dosage of warfarin has been reported to reach 10 mg [7]. Eighty percent of warfarin is excreted in the urine, while the remaining twenty percent is found in the feces (Ufer, 2005). Given that the individual volume of urine excreted per day is on average 1.89 L (1.78–2 L) [8], the concentration of warfarin in human urine will theoretically be equal to 4.23 mg/L (=8/1.89). The lowest warfarin concentration utilized in this study was thus 5 mg/L. According to [9], the incidence of pulmonary thrombosis was 30% in COVID-19 patients, whereas it was only 1.3% for normal individuals, resulting in a ratio of 23.07 (=30/1.3). Subsequently, it was decided to consider a second concentration equal to 25 mg/L that is representative of COVID-19 conditions. Vitamin K antagonists, including warfarin, cannot act rapidly; they must be preceded by heparin treatment, particularly tinzaparin in this context. Consequently, identical concentrations were utilized for both anticoagulants, facilitating subsequent comparisons of their respective toxicities. It should be noted that these concentrations of warfarin and tinzaparin were previously employed by [10] on a community of meiobenthic nematodes from the same site as the investigation in the present research and induced notable taxonomic and functional alterations after a 30-day bioassay. For zebrafish larvae at 17 days post-fertilization [2], among the concentrations of 5 mg/L, 25 mg/L, and 125 mg/L, a significant mortality rate was only observed at the 125 mg/L concentration of warfarin. In this experiment, we focused solely on the concentrations that discernibly affect meiobenthic nematodes, with a comparable biomass to amphipods, and do not exhibit noticeable mortality in zebrafish, specifically 5 mg/L and 25 mg/L. This approach was adopted to expedite the detection of the impacts of this anticoagulant at lower doses and to investigate its effects on meiobenthic amphipods, which are smaller than zebrafish larvae. Warfarin ‘W’ (Sigma-Aldrich, Taufkirchen, Germany) and tinzaparin ‘T’ (Sigma-Aldrich) were administered in prefiltered seawater (0.7 μm, Glass Microfibre GF/F, Whatman, Maidstone, UK) sourced from Al Saif Bay to attain concentrations of 5 mg/L (referred to as W1 and T1) and 25 mg/L (referred to as W2 and T2). This study also included an untreated control group (UC) and four additional combinations: T1W1, T2W2, T1W2, and T2W1. To minimize the degradation of the anticoagulant drugs caused by light exposure and evaporation, all spiking procedures were carried out in a cold (4 °C) and dark environment, thereby limiting any potential handling issues.

### 2.3. Experimental Design

The bioassay employed 1.5 L bottles (microcosms) equipped with plastic caps featuring two openings (refer to Figure 1). The first opening connected each microcosm to an air diffuser to supply oxygen to meiobenthic organisms. The second opening facilitated a continuous release of air to prevent pressure accumulation that could compromise the integrity of the bottles. Each bottle contained 300 g of sediment with its natural meiofauna intact, in addition to one liter of filtered seawater with a pore size of 0.7 μm (Glass Microfibre GF/F, Whatman, Maidstone, UK). Numerous published studies [11,12,13,14] have validated the efficacy of this experimental design. Before the experiment started, all microcosms (containing amphipods) were left in complete darkness at a temperature of 4 °C for one week of acclimatization. Certainly, meiofauna exhibits aggregative segregation in their environment, characterized by distinct assemblages of organisms with higher densities compared to surrounding areas in natural settings [15]. Before adding 300 g into each bottle at the acclimatization start, natural sediment was homogenized using a food mixer, a standard procedure for initiating such experiments in meiobenthology [11,16,17,18,19]. This procedure guarantees an even distribution of meiobenthic organisms and the uniformity of organic matter throughout the sediment column. Previous studies have demonstrated that within one day of post-mixing, meiobenthic organisms tend to redistribute themselves vertically based on their oxygen requirements [20,21]. However, this observation is not applicable in our case due to the sediment layers being only 2–3 cm thick. Indeed, the oxygenation provided by the aquarium air stone is sufficient to saturate the entire microcosm throughout the experiment duration. After the acclimatization phase, the bioassay was carried out for one month under the same laboratory conditions. Each treatment was performed in triplicate (27 microcosms in total), with complete water changes occurring every 24 h to maintain consistent levels of warfarin and tinzaparin within the microcosms.

### 2.4. Meiobenthos Counting and Amphipod Taxonomy (Figure 1)

After the experiment ended, levigation, decantation, and sieving were conducted in five iterations using a combination of stacked sieves (1 mm and 40 μm) to separate meiobenthic organisms from sediment, following the procedure outlined by [22]. The stacked sieves enabled the separation of larger macrofauna from smaller meiofauna, with only the specimens gathered on the lower sieve being kept. Additionally, a small amount of Rose Bengal (0.2 g/L) was added [23,24,25]. This pink staining was essential for subsequent sorting processes using a stereomicroscope Wild-M3B (Wild Heerbrugg AG, Gais, Switzerland).

The Image Software NIS Elements Analysis Version 4.0 (Nikon 4.00.07-build 787-64 bit, Nikon Corporation, Tokyo, Japan) was utilized in conjunction with a Nikon DS-Fi2 camera (Nikon Corporation, Tokyo, Japan) connected to a Nikon microscope to identify all amphipods down to the species level [26,27,28,29,30]. The species naming adhered to the ERMS guidelines [31]. Taxonomic methods based on community composition included the determination of species count (S), Margalef’s richness index (d), the Shannon–Wiener index (H’), and Pielou’s evenness (J’).

### 2.5. Computational Modeling

The toxicokinetic properties of warfarin and tinzaparin were evaluated based on the physicochemical characteristics. Assessments of bioavailability and toxicokinetics were conducted, focusing on the absorption, distribution, metabolism, excretion, and toxicity, as outlined in earlier research studies [3,32,33].

A modeling technique was used to analyze warfarin and tinzaparin binding scores and molecular interactions with a GH7 Family Cellobiohydrolase structure derived from Daphnia. The macromolecule designated as 4XNN was obtained from the RCSB and selected for this study. Both anticoagulants and 4XNN were previously characterized and subsequently examined using a CHARMM force field, following the methodologies described in previous studies [34,35,36].

### 2.6. Data Processing and Statistics

Kolmogorov–Smirnov tests were employed to evaluate the normality of the data, while the Bartlett test was used to assess the equality of variances. Log transformations were applied as necessary. The software STATISTICA version 8 was utilized to perform a one-way analysis of variance (ANOVA) and subsequent Tukey’s HSD tests for significant overall comparisons and multiple comparisons, respectively.

Multivariate analyses were conducted using PRIMER v.5 software from Plymouth Routines, as outlined in studies [37,38]. Non-metric multidimensional scaling (nMDS) was first performed on square root transformed species abundances using the Bray–Curtis similarity matrix to explore taxonomic relationships among treatments. After conducting an ANOSIM test to detect significant taxonomic or functional variations from the control group, SIMPER analysis was employed to evaluate the contribution of specific species or functional groups. A second-stage nMDS was then conducted to integrate all functional characteristics and determine the functional nMDS trait that most accurately corresponds to the nematode taxonomic trait using single-linkage Bray–Curtis similarity.

## 3. Results

### 3.1. Environmental Factors at the Prospected Site

On the day of sampling, the sediment depth was measured at 1 m. The salinity level was recorded at 38.51 PSU, and the temperature was noted as 25.6 °C. Additionally, the dissolved oxygen concentration was measured at 7.13 mg/L. The sediment composition consisted of 22.13 ± 0.09% fine particles and 77.87 ± 0.09% coarse particles, each with a size of 63 μm. Furthermore, the sandy portion exhibited a median grain size of 281 ± 10 μm, and the total organic matter content was calculated to be 6.42 ± 0.17%. Lastly, the water content was measured at 27.46 ± 4.12%.

### 3.2. Meiofaunal Abundances

Table 1 outlines the distributions of meiobenthic communities. In the meiofauna samples collected after the bioassay, free-living nematodes were present across all treatments, regardless of contamination with tinzaparin and warfarin. Alongside nematodes, the meiofauna community after exposure to UC included amphipods, polychaetes, oligochaetes, turbellaria, copepods, gastrotrichs, and tardigrades, listed according to their abundance.

After exposure to tinzaparin and warfarin, the populations of all meiofauna groups decreased over time, with varying responses depending on whether the medications were administered alone or in combination. By the end of the experiment, nematodes and amphipods were the only meiobenthic groups found in every treatment. The other groups were consistently absent following exposure to either the individual or combined treatments, resulting in gaps in Table 1.

Nematodes showed a significant reduction in abundance in treatments T2, T2W1, T1W2, W2, and T1W1 compared to the untreated control group (UC), as indicated by pairwise multiple comparisons (Tukey’s HSD test: *p*-value < 0.05). The variations in amphipod populations exhibited a distinct trend in comparison to other meiobenthic species. Treatment T1 had a substantially higher population of amphipods than UC, whereas treatments T2W1 and T2W2 showed a significant decline relative to the control group (Tukey’s HSD test: *p*-value < 0.05).

### 3.3. Taxonomic Census and Diversity of Amphipods

After the experiment, a total of 11 families, 13 genera, and 16 species of amphipods were identified (see Table 2). The families with the greatest diversity were Aoridae, which had three species, and Ampithoidae and Corophiidae, each with two species. The remaining eight families were represented by only one species each (refer to Table 2). The lowest number of species recorded was seven in the T2 and T2W1 treatments. The treatments T1, T1W1, and T2W2 each contained 10 species. The control treatment showed the highest species diversity, with 15 species identified. *Ampithoe ferox* and *Longigammarus bruni* were the most dominant species, each comprising approximately 15.26% ± 3.98% of the total population.

Several species were prevalent across multiple treatments involving tinzaparin and/or warfarin: *Gammarella fucicola* was found in all treatments except for UC and T2W1; *Leptocheirus hirsutimanus* was present in T2; *Longigammarus bruni* was found in T2, T1W2, and T2W1; *Lysianassina longicornis* was identified in T2W2; *Paraniphargus valesi* appeared in all treatments except for UC; *Microdeutopus algicola* was recorded across T2, W1, W2, T1W1, T2W2, T1W2, and T2W1; *Microdeutopus gryllotalpa* was found in all treatments except for UC and T1; *Siphonoecetes dellavallei* was present in all treatments except for UC, T1, and T2; *Stenothoe monoculoides* was recorded only in T1.

**Figure 2:**
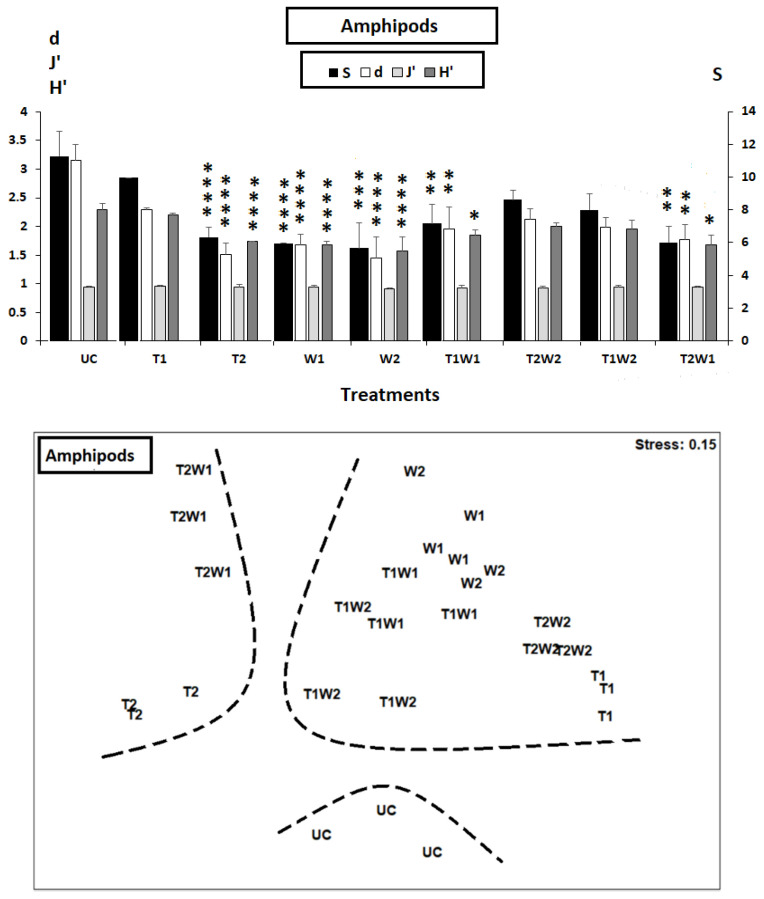
Graphical summary of univariate taxonomic indices (**above**) and non-metric multidimensional scaling 2D plot based on nematode species abundances (**below**) of the control microcosms (UC) and those enriched with two anticoagulant drugs, tinzaparin (T) and warfarin (W), and their mixtures (T1W1, T2W2, T1W2, and T2W1). UC = untreated control; T1 = tinzaparin (5 mg/L), T2 = tinzaparin (25 mg/L), W1 = warfarin (5 mg/L), and W2 = warfarin (25 mg/L). S = species number, d = Margalef’s Species Richness, H’ = Shannon–Wiener index, and J’ = Pielou’s evenness. The stars indicate significant differences compared to the controls (UC) (* = *p* < 0.05; ** = *p* < 0.001; *** = *p* < 0.0001; **** = *p* < 0.00001) (Tukey’s HSD test, *Log*-transformed data).

The taxonomic diversity showed significant changes in the groups treated with tinzaparin and/or warfarin compared to the control group (see Figure 2). Among the intervention groups, UC, T1, T2W2, and T1W2 displayed the highest levels of variation, while T2, W1, W2, T1W1, and T2W1 exhibited the least diversity. Similar trends were observed in Margalef’s species richness and the Shannon index, as indicated by Tukey’s HSD test (*p*-value < 0.001, Figure 2). In contrast, Pielou’s evenness did not show any significant differences across the treatments (1-ANOVA: *p*-value = 0.735, Figure 2).

### 3.4. Multivariate Analyses Based on Community Composition of Amphipods

The non-metric multidimensional scaling (nMDS) analysis (stress = 0.15) of the taxonomic composition of meiobenthic amphipods produced results that aligned closely with the diversity indices (Figure 2). The control group of amphipods was distinctly positioned at the base of the two-dimensional graph. In the ordination space, two separate taxonomic categories emerged: one cluster, representing moderate diversity, was on the right side (including T1, W1, W2, T1W1, T2W2, and T1W2), while the group with the lowest diversity was situated farthest from the control replicates on the left side (comprising T2 and T2W1).

The ANOSIM results indicated significant taxonomic variation between the control group and those treated with tinzaparin, warfarin, or both, with a *p*-value of 0.01 and an *R*-statistic of 0.778 (Table 3).

The SIMPER analysis (Table 3) highlighted that the most distinct amphipod communities, when compared to the control, were T2 and T2W1, which exhibited average dissimilarities of 64.31% and 61.56%, respectively. Table 3 also summarizes the trends, emphasizing the taxa that contributed to approximately 50% of the variance from the untreated control group:

- The species *Stenothoe monoculoides* showed a significant decline at all concentrations of tinzaparin and warfarin, both alone and in combination. This species completely disappeared from six out of the eight examined assemblages: T2, W1, W2, T1W1, T1W2, and T2W1.
- However, exposure to certain treatments was sometimes beneficial for specific species. This was the case for *Longigammarus bruni* (in treatments T1, T2, and T1W2), *Microdeutopus versiculatus* (in treatment T1), and *Leptocheirus pilosus* (in treatment T2).
- In treatments T1 and T2, there was a reduction in the abundance of several amphipods, including *Stenothoe monoculoides*, *Ampithoe ramondi*, and *Microdeutopus gryllotalpa*. Following the introduction of tinzaparin, *Ampithoe ramondi* was completely eradicated in treatment T1, and *Microdeutopus gryllotalpa* vanished from the T2 treatments.
- A notable decrease in the populations of seven species was observed across treatments W1 and W2, which included *Stenothoe monoculoides*, *Ampithoe ramondi*, and *Lysianassina longicornis*. Among these, both *Stenothoe monoculoides* and *Ampithoe ramondi* disappeared from W1 and W2.
- The third pattern emerged in the mixture treatments, where SIMPER analysis revealed that *Lysianassina longicornis* and *Ampithoe ferox* were diminished or eliminated (as shown in Table 3). Furthermore, *Ampithoe ramondi* was eradicated in treatment T2W2, and its relative abundance decreased in treatment T2W1.

### 3.5. Computational Modeling

**Figure 3:**
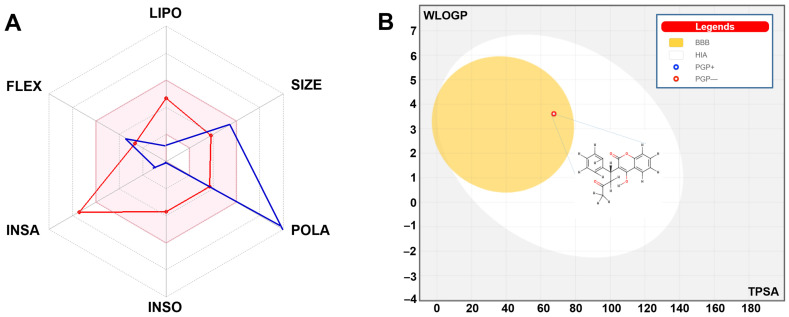
(**A**) Oral bioavailability hexagon of warfarin (red) and tinzaparin (blue) based on the physicochemical properties and (**B**) the corresponding boiled egg model. LIPO, SIZE, POLA, INSO, INSA, and FLEX stand for lipophilicity, molecular size, polarity, insolubility, unsaturation, and flexibility. Note that both warfarin and tinzaparin possessed a good oral bioavailability (in the pink area, (**A**)), while tinzaparin is out of range in the boiled egg model, and warfarin possessed high gastrointestinal (GI) absorption and blood–brain barrier (BBB) permeation (in the yellow area, (**B**)).

Table 4 shows that while warfarin can cross the blood–brain barrier (BBB) and serve as a substrate for glycoprotein (P-gp), tinzaparin was not a BBB permeant but a P-gp substrate. Warfarin has favorable oral bioavailability and significant gastrointestinal (GI) absorption. The results were further supported by the boiled egg model and the bioavailability hexagon (see Figure 3). As a known substrate for P-gp, warfarin has been shown to inhibit the enzymes CYP2C19 and CYP2C9, as indicated by references [3,39]. However, it does not affect CYP1A2, CYP2D6, or CYP3A4. Tinzaparin was predicted to inhibit none of the five major CYP isoforms.

The study calculated the skin permeability coefficient (Log Kp) by considering molecular size and lipophilicity, taking into account the physicochemical properties. The results indicated minimal permeability and average solubility, resulting in a Log Kp value of −6.26 cm/s and −11.50 cm/s for warfarin and tinzaparin, respectively.

Warfarin exhibited favorable binding scores of −4.9 kcal/mol (see Table 5). When bound to the 4XNN pocket, warfarin formed standard hydrogen bonds and electrostatic interactions, complemented by π-π T-shaped interactions, π-Alkyl interactions, and deep embedding (2.386 Å). These interactions involved several essential residues, including LYS227, ASP197, ASP203, ASP405, TRP401, and ILE219. Similarly, tinzaparin interacted with several residues through a rich network of bonds, which includes attractive charge, and a conventional H-Bond associated with many hydrophobic interactions (π-Cation, π-Anion, and π-Sulfur).

## 4. Discussion

This study is among the few that have examined the species diversity of nematodes and amphipods within the context of an ecotoxicological experiment. Additionally, only a limited number of previous studies have utilized multiple meiobenthic groups, typically focusing on nematodes and occasionally including harpacticoids, in field surveys and ecotoxicological research. Furthermore, the effects of tinzaparin and warfarin on meiofauna, both taxonomic and functional, had not been previously investigated.

### 4.1. How Did Meiobenthic Organism Abundance Vary in Response to Stress?

In the latest bioassay, the most commonly found meiobenthic organisms were free-living nematodes. It was observed, however, that their population significantly decreased in sediments polluted with either tinzaparin or warfarin, depending on the specific species. The use of these blood-thinning medications led to a decline in particular groups of meiobenthic organisms, including Tardigrada, polychaetes, oligochaetes, turbellaria, copepods, and gastrotrichs. Among these, nematodes and amphipods were the only organisms that showed a frequency of one out of nine. Previous research on polycyclic aromatic hydrocarbons, heavy metals, and pesticides has indicated that these taxa often experience a decline in numbers during stressful conditions.

The results of the experiments suggest various possible outcomes. A notable change observed in the sediment throughout the treatments was its increased adhesive quality, linked to the drugs adhering to the substrate and the remains of vulnerable species. This increased stickiness significantly limited the natural movements of small benthic organisms, as suggested by other studies. Nematodes, known for their small size and distinctive body composition compared to other annelids and crustaceans such as harpacticoid copepods and amphipods, were found to be the most resilient species.

The decrease in population may be associated with the high number of setae relative to their body surface, particularly in polychaetes and copepods. Unlike other annelids, most polychaetes possess additional appendages known as parapods, which are equipped with numerous setae. Small benthic copepods have branched setae, elongated feathery setae, and many spineless, hairy setae on their bodies. Research suggests that warfarin may influence the movement and reproductive capabilities of polychaetes and copepods by reducing the number of setae they possess.

The presence of amphipods at the end of the experiment across all microcosms and following exposure to all treatments indicated their suitability for further examination. These peracarid crustaceans play a crucial role in benthic communities. They are a vital food source for many small invertebrates, fish, and seabirds, and their widespread distribution can be attributed to their resilience to changing ecological conditions.

In this study, it appears that algal biomass influenced the quantity of amphipods. The nutritional, chemical, and structural properties of algae provide herbivores with a varied and diverse feeding environment. Several studies indicate a positive correlation between amphipod abundance and the presence of microalgae in the same habitat. Benthic diatoms were likely the primary food source when tinzaparin or warfarin was introduced into the microcosms. The relationship between chlorophyll and warfarin is not clearly defined; however, warfarin is known to inhibit the activation of vitamin K. Vitamin K functions as an electron transporter in the photosynthetic membranes of chloroplasts.

### 4.2. How Do Amphipods Taxonomically Respond to Stress?

This research recorded a total of 813 amphipod specimens, resulting in the identification of 16 species belonging to 11 families (see Table 2). The literature indicates that there have been no prior studies in Saudi Arabia focusing on meiobenthic amphipods that provide a comprehensive list of native records for the region. Notably, several of the recorded taxa have previously been collected from the Mediterranean Sea and the Black Sea, where over 400 and 100 species of benthic amphipods, respectively, have been identified [40,41,42,43,44,45].

A reduction in species diversity was observed among the meiobenthic amphipods. This reduction is characterized by low diversity globally, particularly among communities subjected to treatments T2, T2W1, W1, W2, and T1W1. It is important to highlight that the least diverse assemblages were observed after the first two treatments that utilized the highest concentration of tinzaparin (T2). The results (shown in Figure 2) indicated that the decrease in diversity was moderate, particularly following exposure to warfarin alone and treatment T1W1. It seems that treatment T1 alone was insufficient to fully mitigate the harmful effects of W1.

In the non-metric multidimensional scaling (nMDS) ordination plot, the groupings T2 and T2W1 exhibited significant differences, with dissimilarity percentages of 64.31% and 61.56%, respectively, compared to the control group (UC). In contrast, the other treatments, which included combinations of warfarin and tinzaparin (W1, W2, T1, T1W2, T1W1, and T2W2), demonstrated medium dissimilarity from the control group (UC).

The SIMPER analysis (see Table 3) further confirmed the significant impact of tinzaparin and/or warfarin on the amphipod community, as previously indicated by the diversity tools. The sediment samples exposed to tinzaparin revealed negative effects on certain species, highlighting their vulnerability to this anticoagulant. Specifically, the species *Stenothoe monoculoides*, *Ampithoe ramondi*, and *Microdeutopus gryllotalpa* were adversely affected. In contrast, three species demonstrated a tolerance to tinzaparin: *Longigammarus bruni*, *Microdeutopus versiculatus*, and *Leptocheirus pilosus*.

Regarding warfarin, *Stenothoe monoculoides*, *Ampithoe ramondi*, and *Lysianassina longicornis* were identified as negative bioindicators. No evident tolerance was recognized among any of the 16 species assessed with warfarin. This variation in tolerance and sensitivity helps explain why *Ampithoe ramondi* was less favored after exposure to T2W2 and T2W1, while *Longigammarus bruni* was favored following the application of T1W2.

### 4.3. Does Computational Modeling Support Univariate and Multivariate Outcomes?

Table 4 presents information about the physicochemical and toxicokinetic characteristics of warfarin and tinzaparin. While it was expected that warfarin would be able to cross the blood–brain barrier (BBB), and not anticipated to be a substrate for P-glycoprotein (P-gp), tinzaparin was not a BBB permeant but a P-gp substrate. Warfarin is known for its high oral bioavailability and effective absorption within the gastrointestinal (GI) system. These observations were supported by the bioavailability hexagon and the boiled egg model referenced earlier (B) (Figure 3). The latter showed that tinzaparin is out of range regarding the boiled egg model, which indicates further low GI absorption-associated non-permeation of the BBB.

Among the five cytochrome P450 (CYP) enzymes examined, CYP1A2, CYP2D6, and CYP3A4 were not expected to be inhibited by warfarin. In contrast, CYP2C19 and CYP2C9 were found to be inhibited. These inhibitions may result in toxicological effects [3,39], which could be further exacerbated by tinzaparin acting as a substrate for P-gp [3,33]. It has been noted that the inhibition of CYPs and interactions with P-gp can interfere with the transport of compounds, subsequently affecting their metabolism [32,33]. Tinzaparin was predicted to inhibit none of the five major CYPs.

The Log Kp value was calculated based on the physicochemical characteristics of the molecule, including its size and lipophilicity. The results showed that warfarin exhibits low permeability and moderate solubility.

**Figure 4:**
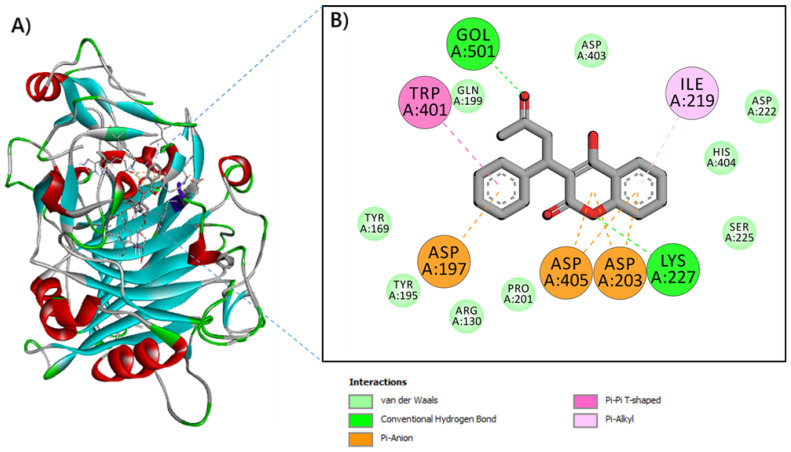
Binding interactions of warfarin and GH7 Family Cellobiohydrolase from *Daphnia* (pdb id: 4XNN). (**A**) Illustration of warfarin docked to the ribbon structure of 4XNN, and (**B**) the resulting 2D diagram of interaction.

In the field of computational biology, the interaction of warfarin with 4XNN was predicted. The results indicated that warfarin displayed negative scores but maintained an acceptable free binding energy of −4.9 kcal/mol (see Table 5). Conventional hydrogen bonds and electrostatic interactions are often evaluated due to their significant roles in biological reactions [3,34,46]. Warfarin exhibited strong interactions with π-π T-shaped and π-alkyl interactions, which were well integrated into the 4XNN pocket at a distance of 2.386 Å (refer to Figure 4). These important interactions involved several essential amino acids, including LYS227, ASP197, ASP203, ASP405, TRP401, and ILE219. Tinzaparin established a rich network of bonds (including attractive charge, conventional H-Bond, π-Cation, etc.) with nine different close interacting residues. It is noteworthy that shallow embedding (distances of less than 2.5 Å) has been associated with significant impacts, as highlighted in sources [35,36]. The findings related to molecular interactions and toxicokinetics support the experimental results and provide deeper insights into the underlying mechanisms. This may also help explain the sensitivity to anticoagulants, particularly among susceptible amphipod taxa.

These computational findings support the potential ecotoxicological effects of warfarin and tinzaparin on meiobenthic amphipods and confirm the interest of combined modeling and experimental approaches.

## 5. Conclusions

The variations in abundance observed in this study indicate that meiobenthic organisms are affected by both warfarin and tinzaparin, the two anticoagulants examined. Among these organisms, nematodes and amphipods were identified as the most resilient groups within the meiofauna community. Notably, the amphipod taxonomic spectrum, previously unexplored in Saudi Arabia, demonstrated a satisfactory level of species diversity along the shores of Jeddah.

Modifications in both abundance and taxonomic diversity were particularly evident in treatments that included T2 (25 mg/L). Interestingly, amphipods exposed to both tinzaparin and warfarin exhibited decreased diversity compared to those receiving T2 and T2W1. This study also showed that the anticoagulants primarily impacted several sensitive amphipod taxa. In contrast, three species tolerant to tinzaparin—*Longigammarus bruni*, *Microdeutopus versiculatus*, and *Leptocheirus pilosus*—were identified as potential positive bioindicators for this anticoagulant.

The computational modeling used in the study provided a clearer understanding of the interactions between the anticoagulants and meiobenthic amphipods. Overall, the results reinforced the value of amphipods as effective bioindicators, especially in the context of specific anticoagulants.

## Figures and Tables

**Table 1:** Abundances of meiobenthic taxa from control microcosms (UC) and those enriched with two anticoagulant drugs, tinzaparin (T) and warfarin (W), and their mixtures (T1W1, T2W2, T1W2, and T2W1). UC = untreated control; T1 = tinzaparin (5 mg/L), T2 = tinzaparin (25 mg/L), W1 = warfarin (5 mg/L), and W2 = warfarin (25 mg/L). Significant differences in comparison to controls using Tukey’s test: *p* < 0.05 (*), *p* < 0.01 (**), *p* < 0.001 (***), and *p* < 0.0001 (****). *log*(*x*)-transformed data (nematodes and amphipods), *log*(*x*+1)-transformed data (remaining taxa).

Taxa	UC	T1	T2	W1	W2	T1W1	T2W2	T1W2	T2W1
Nematodes	606 ± 43	411 ± 67 (*)	172 ± 59 (****)	538 ± 72	345 ± 26 (***)	419 ± 37 (*)	616 ± 54	322 ± 40 (***)	206 ± 32 (****)
Amphipods	27 ± 6	51 ± 3 (***)	34 ± 2	21 ± 7	25 ± 7	26 ± 2	37 ± 2 (*)	34 ± 7	17 ± 4 (***)
Polychaetes	31 ± 3	17 ± 5 (**)	5 ± 2 (****)	21 ± 4 (*)	13 ± 4 (**)	26 ± 5	17 ± 2 (**)	18 ± 3 (**)	
Oligochaetes	29 ± 10	17 ± 3 (**)		8 ± 5 (***)	4 ± 4 (****)		8 ± 2 (***)	15 ± 3 (**)	2 ± 3 (****)
Turbellaria	22 ± 8	23 ± 3	2 ± 1 (****)	5 ± 6 (***)		12 ± 4 (**)	16 ± 5 (*)	7 ± 2 (***)	4 ± 0 (****)
Copepods	19 ± 4	3 ± 0 (****)		9 ± 4 (**)	2 ± 1 (****)	7 ± 5 (**)	6 ± 1 (**)		
Tardigrada	9 ± 2			3 ± 4 (**)	7 ± 2			3 ± 0 (**)	
Gastrotricha	10 ± 2	5 ± 1 (**)		3 ± 1 (***)	8 ± 3	9 ± 3	4 ± 4 (*)	2 ±1 (****)	1 ± 1 (****)

**Table 2:** Average relative abundances of amphipod species (±SD) identified in control microcosms (UC) and those enriched with two anticoagulant drugs, tinzaparin (T) and warfarin (W), and their mixtures (T1W1, T2W2, T1W2, and T2W1). UC = untreated control; T1 = tinzaparin (5 mg/L), T2 = tinzaparin (25 mg/L), W1 = warfarin (5 mg/L), and W2 = warfarin (25 mg/L).

Order	Family	Species	UC	T1	T2	W1	W2	T1W1	T2W2	T1W2	T2W1
Amphipoda	Ampithoidae	*Ampithoe ferox*	15.26 ± 3.98	7.88 ± 5.35		2.08 ± 3.61	2.38 ± 4.12	1.28 ± 2.22	1.96 ± 3.40		
Amphipoda	Ampithoidae	*Ampithoe ramondi*		9.32 ± 4.86				5.24 ± 5.84	2.73 ± 0.18	1.63 ± 2.82	
Amphipoda	Corophiidae	*Apocorophium acutum*	1.04 ± 1.80			2.30 ± 3.98	1.19 ± 2.06				
Amphipoda	Dexaminidae	*Dexamine spinosa*	3.70 ± 6.42		9.76 ± 4.41			1.28 ± 2.22		1.23 ± 2.14	20.03 ± 7.79
Amphipoda	Nuuanuidae	*Gammarella fucicola*	7.53 ± 3.32	11.15 ± 5.16	15.65 ± 9.41	15.92 ± 3.68	16.03 ± 14.13	14.06 ± 1.46	15.33 ± 3.35	10.80 ± 6.80	
Amphipoda	Corophiidae	*Leptocheirus hirsutimanus*	4.17 ± 7.22		16.47 ± 6.17			7.54 ± 7.18		1.96 ± 3.40	
Amphipoda	Gammaridae	*Longigammarus bruni*	15.26 ± 3.98		20.64 ± 5.33			1.39 ± 2.41		19.06 ± 7.15	11.37 ± 3.66
Amphipoda	Lysianassidae	*Lysianassina longicornis*	3.13 ± 5.41	7.19 ± 1.10			1.11 ± 1.92		10.06 ± 9.33		
Amphipoda	Maeridae	*Maera grossimana*	6.53 ± 7.56	6.57 ± 1.40		1.96 ± 3.40			4.39 ± 4.02		
Amphipoda	Melitidae	*Paraniphargus valesi*	6.49 ± 2.92	11.65 ± 4.80	19.89 ± 10.14	23.79 ± 13.58	10.83 ± 0.89	12.58 ± 4.85	11.61 ± 6.34	11.60 ± 5.36	11.69 ± 10.85
Amphipoda	Aoridae	*Microdeutopus algicola*	7.92 ± 4.23	7.92 ± 2.33	16.54 ± 5.29	14.86 ± 7.61	23.43 ± 12.72	23.09 ± 10.01	15.63 ± 5.03	13.43 ± 8.24	23.61 ± 2.73
Amphipoda	Aoridae	*Microdeutopus gryllotalpa*	5.10 ± 2.16	14.23 ± 7.41		22.14 ± 2.95	20.80 ± 2.89	13.95 ± 5.46	16.72 ± 9.32	16.93 ± 2.04	18.90 ± 12.76
Amphipoda	Aoridae	*Microdeutopus versiculatus*	8.96 ± 2.48							6.19 ± 5.38	1.52 ± 2.62
Amphipoda	Atylidae	*Nototropis massiliensis*	6.33 ± 4.22							1.63 ± 2.82	
Amphipoda	Ischyroceridae	*Siphonoecetes dellavallei*	7.53 ± 3.32	8.47 ± 0.68	1.04 ± 1.80	16.95 ± 4.14	24.23 ± 12.47	19.60 ± 7.75	14.45 ± 3.51	15.53 ± 1.34	12.89 ± 5.53
Amphipoda	Stenothoidae	*Stenothoe monoculoides*	1.04 ± 1.80	15.62 ± 4.98					7.12 ± 3.85		

**Table 3:** Amphipod species accounting for ~50% of overall dissimilarity between control microcosms (UC) and those enriched with two anticoagulant drugs, tinzaparin (T) and warfarin (W), and their mixtures (T1W1, T2W2, T1W2, and T2W1) based on outcomes of similarity percentage analysis ‘SIMPER’ (square root transformed abundances). UC = untreated control; T1 = tinzaparin (5 mg/L), T2 = tinzaparin (25 mg/L), W1 = warfarin (5 mg/L), and W2 = warfarin (25 mg/L).

Comparisons	UC vs. T1	UC vs. T2	UC vs. W1	UC vs. W2
R-statistics	1	1	0.926	1
*p*-value	0.01	0.01	0.01	0.01
AD (%)	47.87	64.31	40.89	41.23
	*Stenothoe monoculoides* (13.44%) −	*Stenothoe monoculoides* (12.01%) Ø	*Stenothoe monoculoides* (20.97%) Ø	*Stenothoe monoculoides* (20.42%) Ø
	*Ampithoe ramondi* (11.39%) Ø	*Longigammarus bruni* (11.29%) +	*Ampithoe ramondi* (15.81%) Ø	*Ampithoe ramondi* (15.40%) Ø
	*Longigammarus bruni* (10.72%) +	*Microdeutopus gryllotalpa* (11.25%) Ø	*Lysianassina longicornis* (14.32%) Ø	*Maera grossimana* (13.30%) Ø
	*Microdeutopus versiculatus* (8.21%) +	*Leptocheirus pilosus* (10.04%) +		*Lysianassina longicornis* (11.62%) −
	*Microdeutopus gryllotalpa* (8.02%) −	*Ampithoe ramondi* (9.05%) Ø		
**Comparisons**	**UC vs. T1W1**	**UC vs. T2W2**	**UC vs. T1W2**	**UC vs. T2W1**
R-statistics	1	0.963	0.778	0.889
*p*-value	0.01	0.01	0.01	0.01
AD (%)	40.71	20.38	47.26	61.56
	*Stenothoe monoculoides* (19.50%) Ø	*Ampithoe ferox* (18.24%) −	*Stenothoe monoculoides* (15.65%) Ø	*Stenothoe monoculoides* (14.32%) Ø
	*Lysianassina longicornis* (13.32%) Ø	*Stenothoe monoculoides* (15.37%) −	*Longigammarus bruni* (13.94%) +	*Gammarella fucicola* (11.96%) Ø
	*Maera grossimana* (12.70%) Ø	*Ampithoe ramondi* (13.92%) −	*Ampithoe ferox* (10.81%) Ø	*Ampithoe ramondi* (10.80%) Ø
	*Ampithoe ferox* (11.18%) −	*Lysianassina longicornis* (11.43%) st	*Lysianassina longicornis* (10.69%) Ø	*Ampithoe ferox* (9.88%) Ø
				*Lysianassina longicornis* (9.78%) Ø

**Table 4:** Physicochemical properties, toxicokinetics, and toxicity prediction of warfarin and tinzaparin.

	Warfarin	Tinzaparin
Physicochemical properties		
Molecular weight (g × mol^−1^)	303.33	458.39
Num. of heavy atoms	23	31
Num. of arom. heavy atoms	16	0
Fraction of Csp3	0.16	0.88
Num. of rotatable bonds	4	5
Num. of H-bond acceptors	4	15
Num. of H-bond donors	1	8
Molar refractivity	88.58	92.52
TPSA (Å^2^)	67.51	232.24
Water solubility/lipophilicity		
Log S (ESOL)	−3.70	−0.22
Log S (Ali)	−3.77	−0.91
Log S (SILICOS-IT)	−6.33	4.49
Consensus Log *P*_o/w_	3.12	−4.17
Oral toxicity and toxicokinetics		
GI absorption	High	Low
BBB permeant	Yes	No
P-gp substrate	No	Yes
CYP1A2 inhibitor	No	No
CYP2C19 inhibitor	Yes	No
CYP2C9 inhibitor	Yes	No
CYP2D6 inhibitor	No	No
CYP3A4 inhibitor	No	No
Log Kp (cm/s)	−6.26	−11.50

**Table 5:** Binding affinity, conventional hydrogen bonds, and the closest interacting residues of GH7 Family Cellobiohydrolase from *Daphnia* (pdb id: 4XNN) with warfarin anticoagulant.

	Affinity (Kcal/Mol)	Molecular Interactions	
		Closest Interacting Residues	No. of Closest Interacting Residues (Å)
Warfarin	−4.9	Conventional H-Bond: LYS227 (2.386), GOL501 (2.504)	7
		π-Anion (Electrostatic): ASP197 (3.983), ASP203 (3.507), ASP203 (3.708), ASP405 (3.375), ASP405 (4.189)	
		π-π T-shaped: TRP401 (5.023)	
		π-Alkyl: ILE219 (5.015)	
Tinzaparin	−7.6	Attarctive Charge: ASP403 (2.88), ASP405 (2.607)	9
		Conventional H-Bond: ARG130 (2.447), TYR169 (1.967), ARG275 (2.460), TYR195 (3.335)	
		π-Cation: TRP410 (3.818)	
		π-Anion: TRP410 (4.785), TRP401 (3.849)	
		π-Sulfur: TRP410 (4.427), TYR195 (5.28), HIS252 (5.393)	

## Data Availability

All the data in the article are available from the corresponding author upon reasonable request.

## References

[1] Chen J., Du Z., Song B., Li R., Jia X., Chen J., Liu X., Zhong S.A. (2022). A Natural Heparinoid from Mollusc Meretrix Lusoria: Purification, Structural Characterization, and Antithrombotic Evaluation. Curr. Res. Food Sci..

[2] Fernández I., Santos A., Cancela M.L., Laizé V., Gavaia P.J. (2014). Warfarin, a Potential Pollutant in Aquatic Environment Acting through Pxr Signaling Pathway and γ-Glutamyl Carboxylation of Vitamin K-Dependent Proteins. Environ. Pollut..

[3] Hong J.H., Semprucci F., Raehyuk J., Kim K., Lee S., Jeon D., Yoo H., Kim J., Kim J., Yeom J. (2020). Meiobenthic nematodes in the assessment of the relative impact of human activities on coastal marine ecosystems. Environ. Monit. Assess..

[4] Semprucci F., Cesaroni L., Guidi L., Balsamo M. (2018). Do the Morphological and Functional Traits of Free-Living Marine Nematodes Mirror Taxonomical Diversity?. Mar. Environ. Res..

[5] Flumignan R.L., Civile V.T., de Sá Tinôco J.D., Pascoal P.I., Areias L.L., Matar C.F., Tendal B., Trevisani V.F., Atallah Á.N., Nakano L.C. (2020). Anticoagulants for People Hospitalised with COVID-19. Cochrane Database Syst. Rev..

[6] Neely J.L., Carlson S.S., Lenhart S.E. (2002). Tinzaparin Sodium: A Low-Molecular-Weight Heparin. Am. J. Health-Syst. Pharm..

[7] Harrison L., Johnston M., Massicotte M.P., Crowther M., Moffat K., Hirsh J. (1997). Comparison of 5-Mg and 10-Mg Loading Doses in Initiation of Warfarin Therapy. Ann. Intern. Med..

[8] Schoen T., Blum J., Paccaud F., Burnier M., Bochud M., Conen D. (2013). Factors Associated with 24-Hour Urinary Volume: The Swiss Salt Survey. BMC Nephrol..

[9] Komiyama M., Hasegawa K. (2020). Anticoagulant Therapy for Patients with Coronavirus Disease 2019: Urgent Need for Enhanced Awareness. Eur. Cardiol. Rev..

[10] Bin-Jumah M.N. (2024). Are Anticoagulant Drugs Ecotoxic for Meiobenthic Nematodes from Saudi Arabia? First Data on Taxon/Functional Diversity and Computational Evidences. Mar. Pollut. Bull..

[11] Austen M.C., McEvoy A.J., Warwick R.M. (1994). The Specificity of Meiobenthic Community Responses to Different Pollutants: Results from Microcosm Experiments. Mar. Pollut. Bull..

[12] Hubas C., Sachidhanandam C., Rybarczyk H., Lubarsky H., Rigaux A., Moens T., Paterson D. (2010). Bacterivorous nematodes stimulate microbial growth and exopolymer production in marine sediment microcosms. Mar. Ecol. Prog. Ser..

[13] Armenteros M., Pérez-García J.A., Ruiz-Abierno A., Díaz-Asencio L., Helguera Y., Vincx M., Decraemer W. (2010). Effects of organic enrichment on nematode assemblages in a microcosm experiment, Mar. Environ. Res..

[14] Hermi M., Mahmoudi E., Beyrem H., Aïssa P., Essid N. (2009). Responses of a free-living marine nematode community to mercury contamination: Results from microcosm experiments. Arch. Environ. Contam. Toxicol..

[15] Moccia D., Cau A., Meloni M.C., Pusceddu A. (2019). Small-Scale Distribution of Metazoan Meiofauna and Sedimentary Organic Matter in Subtidal Sandy Sediments (Mediterranean Sea). Adv. Oceanogr. Limnol..

[16] Gyedu-Ababio T.K., Baird D. (2006). Response of Meiofauna and Nematode Communities to Increased Levels of Contaminants in a Laboratory Microcosm Experiment. Ecotoxicol. Environ. Saf..

[17] Louati H., Ben Said O., Soltani A., Cravo-Laureau C., Preud’Homme H., Duran R., Aissa P., Mahmoudi E., Pringault O. (2014). Impacts of bioremediation schemes for the mitigation of a low-dose anthracene contamination on free-living marine benthic nematodes. Ecotoxicology.

[18] Ben Said O., Louati H., Soltani A., Preud’homme H., Cravo-Laureau C., Got P., Pringault O., Aissa P., Duran R. (2015). Changes of benthic bacteria and meiofauna assemblages during bio-treatments of anthracene-contaminated sediments from Bizerta lagoon (Tunisia). Environ. Sci. Pollut. Res..

[19] Schratzberger M., Warwick R.M. (1998). Effects of the Intensity and Frequency of Organic Enrichment on Two Estuarine Nematode Communities. Mar. Ecol. Prog. Ser..

[20] Boufahja F., Semprucci F., Beyrem H. (2016). An Experimental Protocol to Select Nematode Species from an Entire Community Using Progressive Sedimentary Enrichment. Ecol. Indic..

[21] Boufahja F., Semprucci F. (2015). Stress-Induced Selection of a Single Species from an Entire Meiobenthic Nematode Assemblage: Is This Possible Using Iron Enrichment and Does Pre-Exposure Affect the Ease of the Process? Environ. Sci. Pollut. Res..

[22] Vitiello P., Dinet A. (1979). Définition et Échantillonnage Du Méiobenthos. Rapp. Comm. Int. Mer Médit..

[23] Bin-Jumah M.N. (2023). Do functional traits and biochemical biomarkers of the nematode *Oncholaimus campylocercoides* De Coninck and Schuurmans Stekhoven, 1933 affected by fluoranthene and polystyrene microplastics? Results from a microcosm bioassay and molecular modeling. Mar. Pollut. Bull..

[24] Grego M., Stachowitsch M., De Troch M., Riedel B. (2013). CellTracker Green labelling vs. rose bengal staining: CTG wins by points in distinguishing living from dead anoxia-impacted copepods and nematodes. Biogeosciences.

[25] Guo Y., Somerfield P.J., Warwick R.M., Zhang Z. (2001). Large-Scale Patterns in the Community Structure and Biodiversity of Freeliving Nematodes in the Bohai Sea, China. J. Mar. Biol. Assoc. U. K..

[26] Bellan Santini D., Karaman G., Krapp-Schickel G., Ledoyer M., Myers A., Ruffo S., Schiecke U. (1998). The Amphipoda of the Mediterranean.

[27] Bellan Santini D., Diviacco G., Krapp-Schickel G., Myers A., Ruffo S., Ruffo S. (1989). Gammaridea (Haustoriidae to Lysianassidae). The Amphipoda of the Mediterranean, Part II.

[28] Bellan Santini D., Karaman G., Krapp-Schickel G., Ledoyer M., Myers A., Ruffo S., Ruffo S. (1989). Gammaridae (Melphidippidae to Talitridae) Ingolfiellidae, Caprellidae. The Amphipoda of the Mediterranean, Part III.

[29] Bellan Santini D., Karaman G., Krapp-Schickel G., Ledoyer M., Myers A., Ruffo S., Schiecke U., Ruffo S. (1982). Gammaridea (Acanthonozomatidae to Gammaridae). The Amphipoda of the Mediterranean, Part I.

[30] d’Acoz U., Vader W. (2005). The Mediterranean Bathyporeia Revisited (Crustacea, Amphipoda, Pontoporeiidae), with the Description of a New Species. Boll. Mus. Civ. Stor. Nat. Verona.

[31] ERMS European Register of Marine Species. https://www.marbef.org/data/erms.php.

[32] Alreshidi M., Abdulhakeem M.A., Badraoui R., Amato G., Caputo L., De Martino L., Nazzaro F., Fratianni F., Formisano C., De Feo V. (2023). *Pulicaria incisa* (Lam.) DC. as a Potential Source of Antioxidant, Antibacterial, and Anti-Enzymatic Bioactive Molecules: Phytochemical Constituents, in Vitro and in Silico Pharmacological Analysis. Molecules.

[33] Bédoui I., Nasr H.B., Ksouda K., Ayadi W., Louati N., Chamkha M., Choura S., Gargouri J., Hammami S., Affes H. (2024). Phytochemical Composition, Bioavailability and Pharmacokinetics of Scorzonera Undulata Methanolic Extracts: Antioxidant, Anticancer, and Apoptotic Effects on MCF7 Cells. Pharmacogn. Mag..

[34] Akacha A., Badraoui R., Rebai T., Zourgui L. (2022). Effect of Opuntia Ficus Indica Extract on Methotrexate-Induced Testicular Injury: A Biochemical, Docking and Histological Study. J. Biomol. Struct. Dyn..

[35] Ben Saad H., Frikha D., Bouallegue A., Badraoui R., Mellouli M., Kallel H., Pujo J.M., Ben Amara I. (2023). Mitigation of Hepatic Impairment with Polysaccharides from Red Alga *Albidum corallinum* Supplementation through Promoting the Lipid Profile and Liver Homeostasis in Tebuconazole-Exposed Rats. Pharmaceuticals.

[36] Rahmouni F., Hamdaoui L., Saoudi M., Badraoui R., Rebai T. (2024). Antioxidant and Antiproliferative Effects of *Teucrium polium* Extract: Computational and in Vivo Study in Rats. Toxicol. Mech. Methods.

[37] Clarke K.R. (1993). Non-parametric Multivariate Analyses of Changes in Community Structure. Aust. J. Ecol..

[38] Clarke K.R., Gorley R.N. (2001). PRIMER v5: User Manual/Tutorial, PRIMER-E, Plymouth UK. Bull. Mar. Coast. Res..

[39] Othman I.M., Gad-Elkareem M.A., Radwan H.A., Badraoui R., Aouadi K., Snoussi M., Kadri A. (2021). Synthesis, Structure-Activity Relationship and in silico Studies of Novel Pyrazolothiazole and Thiazolopyridine Derivatives as Prospective Antimicrobial and Anticancer Agents. ChemistrySelect.

[40] Bakir K., Sezgin M., Katagan T. (2010). Alien Amphipods on the Turkish Coasts. Zool. Baetica.

[41] Bakir K., Katağan T., Sezgin M. (2008). Parhyale Explorator Arresti, 1989 (Amphipoda, Talitroidea): First Mediterranean Record of This Atlantic Amphipod. Crustaceana.

[42] Bellan-Santini D., Ruffo S. (2003). Biogeography of Benthic Marine Amphipods in Mediterranean Sea. Biogeogr. J. Integr. Biogeogr..

[43] Greze I.I. (1977). Amphipods of the Black Sea and Its Biology.

[44] Mülayim A., Balkis H., Sezgin M. (2015). Benthic Amphipod (Crustacea) Fauna of the Bandırma and Erdek Gulfs and Some Environmental Factors Affecting Their Distribution. Acta Adriat..

[45] Sezgin M., Katağan T. (2007). An Account of Our Knowledge of the Amphipod Fauna of the Black Sea. Crustaceana.

[46] Badraoui R., Adnan M., Bardakci F., Alreshidi M.M. (2021). Chloroquine and Hydroxychloroquine Interact Differently with ACE2 Domains Reported to Bind with the Coronavirus Spike Protein: Mediation by ACE2 Polymorphism. Molecules.

